# Prevalence of Insomnia Symptoms and Need for an Intervention Among Direct-Care Workers in Long-Term Care

**DOI:** 10.1093/geroni/igaa057.2456

**Published:** 2020-12-16

**Authors:** Soomi Lee, Taylor Vigoureux, Kathryn Hyer, Brent Small

**Affiliations:** University of South Florida, Tampa, Florida, United States

## Abstract

This study examined the prevalence of insomnia symptoms among direct-care workers at an assisted-living community and their perceived need for a sleep intervention. Thirty-five participants reported their main sleep-related concerns, willingness to participate in a sleep intervention, and preferred delivery forms/content of the intervention. They also reported nightly sleep characteristics via ecological momentary assessment (EMA) for 2 weeks. 80% reported any sleep-related concern; insomnia-related concern was most prevalent (57%). This was also evident in their EMA reports of waking up in the middle of the night or early morning for 72% of the days. Most (66%) expressed interest in participating in a sleep intervention either online or in group sessions. Mindfulness strategies were most preferred, followed by cognitive-behavioral therapy, and sleep hygiene education. The high prevalence of insomnia symptoms in direct-care workers needs to be addressed by future interventions for their well-being as well as for the quality of care.

